# Anthropometric Assessment of Nepali Children Institutionalized in Orphanages

**DOI:** 10.3390/children7110217

**Published:** 2020-11-07

**Authors:** Lucía Fernández, Ana Rubini, Jose M. Soriano, Joaquín Aldás-Manzano, Jesús Blesa

**Affiliations:** 1 Food & Health Lab, Institute of Materials Science, University of Valencia, 46980 Paterna, Spain; lucia.fernandez.mol@gmail.com (L.F.); Rubinigimenez@gmail.com (A.R.); jesus.blesa@uv.es (J.B.); 2 Joint Research Unit on Endocrinology, Nutrition and Clinical Dietetics, University of Valencia-Health Research Institute La Fe, 46026 Valencia, Spain; 3 Department of Marketing and Market Research, Faculty of Economics, University of Valencia, 46022 Valencia, Spain; joaquin.aldas@uv.es; 4 Laboratory of Nutrition, Department of Preventive Medicine and Public Health, Faculty of Pharmacy, University of Valencia, 46100 Burjassot, Spain

**Keywords:** orphanage, Nepal, anthropometry

## Abstract

Nepal is among the world’s poorest countries, and it is the third-poorest country in the South Asian region. Asia has the largest number of orphans in the world; in Nepal there are around 13,281 orphan children. The objective of this study is to evaluate the growth status of institutionalized children in Nepal through the analyses of anthropometric measures. The sample was Nepalese children aged 4 to 17, obtained from two different orphanages: in the first one, children with physical and mental disabilities coexist with children without any conditions. In the second one, there were no subjects with disabilities. Significant evidence of an association between mental and physical disability in institutionalized children and undernutrition (wasting and stunting) was found in this study. There is also weak but significant evidence of a relationship between underweight and being male. The study could help reaching a better understanding of growth status of institutionalized children in Nepal.

## 1. Introduction

In the Asian nation of Nepal, with a population of around 23 million, divided into more than 100 ethnic groups and distributed in 75 different territorial districts, 80% of the population have a sustenance model based on agriculture [1]. In the South Asia region, Nepal is the third-poorest country [2]. Despite being a state where the poverty affects a high percentage of the population, and where some geographic areas could be hostile and even compromise the subsistence of its inhabitants [3], life expectancy is between 67–69 years. It should also be noted that the 35% of the people are under 15 years old and just 7.6% corresponds to people over 60 years of age [4]. On the other hand, Asia has around 60 million children who to have lost one or both parents, being one of the countries in the world with the largest number of orphans [5]. There is no official published government data on the number of children’s homes in Nepal since the country currently lacks a complete data-collection system on children in institutional care [6]. However, according to the Central Children Welfare Council, there are around 13,281 abandoned and orphaned children in Nepal, distributed in 610 orphanages [7]. Despite the fact that the majority of orphaned (or abandoned) children in Nepal are cared for in institutions, they would not be excluded from risks generated by poverty, which reflects the need of authorities to be more concerned and proactive to combat this situation. People with disabilities in Nepal face higher incidence of human rights violations, especially discrimination, unequal treatment, disregard for difference, denial of accessibility, and exclusion [8]. Therefore, a child with mental disability living in an institution, would inevitably have greater difficulty attaining an acceptable quality of life. The aim of this study was to evaluate the growth status of children living in Nepali orphanages through the analysis of anthropometric parameters.

## 2. Materials and Methods

### 2.1. Participants

Research was conducted in the Nepali town of Hetauda, home of the administrative headquarters of Makwanpur district, placed in the central region of Nepal. This territorial district is mostly rural and is administratively divided in 43 rural and municipal development committees and has a population of around 400,000 [9]. This descriptive study was carried out in collaboration with a Spanish non-governmental organization (NGO) for a one-month period during the summer in 2013 according to the volunteering program of the NGO. The sample was 47 Nepali children (24 girls and 23 boys), who were aged from 4 to 17 years. The sample was taken from two different orphanages: the first one was called Bal Griha 1, where children with physical and mental disabilities coexist with children without any conditions (*n* = 24); the second one, called Bal Griha 2, there were no subjects with disabilities (*n* = 23). In the Bal Griha 1, 12 of the 24 children had no disabilities. The rest of the children of this orphanage had different types of disabilities including burn contractures, posttraumatic amputations, neuromuscular alterations, one cerebral palsy, and different degrees of mental retardation. All subjects gave their informed consent for inclusion before they participated in the study. The study was conducted in accordance with the Declaration of Helsinki, and the protocol was approved by the Ethics Committee of University of Valencia (Spain) (EH-1-2012). The primary caregiver (no aides), who had primary responsibility for children, was informed about the study goals and verbal consent was obtained to take their child’s anthropometric measurements.

### 2.2. Anthropometric Measures

Weight and standing height measurements were performed in triplicate and done according to the Anthropometric Standardization Reference Manual [10]. The subjects wore no shoes and light clothes, and all the measurements were taken by the same trained anthropometrist. A Plenna scale (model MEA 07 400, Plenna^®^, São Paulo, SP, Brazil; accuracy of 100 g) was used to determine weight and a Seca stadiometer (model 208, Seca, GmbH, Hamburg, Germany; accuracy of 0.5 cm) was used to determine height. Height-for-age Z-scores (HAZ), weight-for-age Z-scores (WAZ) and body mass index (BMI) for age (BAZ) were calculated using the WHO AnthroPlus software developed by the World Health Organization (http://www.who.int/growthref/tools/en), which uses the 2007 WHO reference values [11]. The stunting and underweight are calculated by Z-score: children with a HAZ, WAZ, or BAZ < −2.0 are classified as stunted, underweight, or thin, respectively [11,12]. A low HAZ indicates an incapacity to reach linear growth potential as a result of health and/or nutritional deficient conditions, which is known as stunted growth [13]. Children with a measurement of <−2 standard deviation (SD) from the median of the reference group were classified as short for their age (stunted), while children with measurement of <−3 SD from the median of the reference group were considered to be severely stunted [12]. A low WAZ is considered to indicate whether a subject is underweight (in the absence of wasting). Lastly, BMI is an important index for thinness in children over 10 years of age since weight-for-age can be calculated only for children aged <10 years [13]. To estimate body mass in children with a lack of limbs or other body components (leg, foot, or hand), equations for the assessment of weights status of amputees were used [14,15,16].

### 2.3. Statistical Analysis

Values for WAZ, HAZ, and BAZ are presented as means and SD. These means are grouped by sex, orphanage, physical disability, and mental disability. A Kolmogorov–Smirnov test was used to test for normality. To compare the means, the Independent-Samples *t*-Test was used in case of a normal distribution, otherwise the Mann–Whitney U test was used. All statistical significance was established at a *p*-value < 0.05. All statistical analyses were performed using the SPSS Statistics 19.0 (SPSS Inc., Chicago, IL, USA).

## 3. Results

The mean age of all enrolled children into the study was 10.4 ± 3.4 years (range: 4–17 years). The mean of the height was 128.7 ± 18.7 cm and the mean of the weight was 26 ± 10.1 kg. Mean of WAZ, HAZ, and BAZ were all negative (−1.9, −1.9, and−1.4, respectively) (Table 1). This indicates that the average of children was smaller and lighter than the growth standard.

The prevalence of undernutrition in orphan or abandoned children is the following: 40.4% (total *n* = 47) were considered stunted, 36% (total *n* = 25) were underweight and 29.8% (total *n* = 47) were wasting for children over age 10. The sample used by the underweight indicator is 25 instead of the total (47) because WAZ can be calculated only for children aged < 10 years [16].

Table 2 shows values for WAZ, HAZ, and BAZ presented as means grouped by several parameters as are orphanage, physical disability, mental disability and sex. In the Bal Griha 1, two children had overlapping physical and mental disability.

For orphanage (Table 2), WAZ and BAZ were not significantly different between two groups. HAZ was significantly lower in the Bal Griha 1 (*t* = −3.47, *p* < 0.01). The mean of HAZ (−2.03) and WAZ (−2.43), which classifies classify the children of Bal Griha 1 as stunted and underweight, respectively.

For physical disability (Table 2), BAZ and WAZ were not significantly different between the two groups. Despite this, it should be noted that the mean of WAZ (−2.90) classifies the children as underweight. HAZ was significantly lower in the children with a physical disability (*t* = 3.94, *p* < 0.01). The mean of HAZ (−3.22) in children with a physical disability is a value below –3.0, which classifies them as severe stunted (moderate and severe based on <−2 Z and <−3 Z score, respectively).

For mental disability (Table 2), HAZ and WAZ were not significantly different between the two groups. Despite this, it should be noted that the mean of HAZ (−2.62) and WAZ (−2.92) classifies the two groups as stunted and underweight, respectively. BAZ was significantly lower in the children with a mental disability (*t* = 2.53, *p* < 0.05). The mean of BAZ (−2.70) in children with mental disabilities classifies them as wasted.

For sex (Table 2), HAZ and BAZ were not significantly different between the two groups. WAZ was significantly lower in the boys (*t* = 2.62, *p* < 0.05). The mean of WAZ (−2.37) classifies the boys as underweight.

The principal strength of this study is reflected that is the first study carried out in Nepali orphanages. On the other hand, principal limitation is the difficulty in accessing in other orphanages.

## 4. Discussion

The analysis of nutritional status of Nepalese children as assessed from WAZ, HAZ, and BAZ showed that undernutrition prevailed in these children compared to the international standard. These institutionalized children had high prevalence of stunting (40.4%), being underweight (36%), and wasting (29.8%). Similar figures were reported in 2011 from the Nepal Demographic Health Survey, where 41% of Nepalese children under five years of age were stunted, 11% were wasted, and 29% were underweight, maintaining that child undernutrition in Nepal is still among the highest in the world [16].

The higher prevalence of wasting found in the present study (29.8%) compared with the data (11%) reported on 2011, could be explained by the fact that our sample includes children with disabilities, who seem to have higher risk of suffering from wasting as supported in our findings; these show that BAZ was significantly lower in the children with a mental disability (*t* = 2.53, *p* < 0.05). This mean of BAZ (−2.70) classifies the children as wasted. Related to this, a study with children with mental disabilities conducted in Turkey showed a decrease in the muscle and fat storages in 38.8% of the sample [17]. Similar results were found in the Egyptian children with mental disabilities [18]. Both research groups ensure to mention that thin musculature indicates low protein and caloric reserve, this could be related with institution living, or could be due to wasting, poor development or both. Even though studies that compare the prevalence of wasting between children with mental and non-mental disabilities were not found, neurologically disabled children are known to be at high risk for developing malnutrition [18]. This could be due to an inadequate nutrient intake produced due to feeding problems or poor feeding knowledge among care providers [19]. In fact, the presence of their poor nutritional status may, in some measure, explain the growth delay commonly manifested in such children [20]. This could also explain that, in the present study, the mean of HAZ of children with mental disabilities (−2.62) classifies them as stunted and the mean of WAZ (2.92) classifies them as underweight, even when HAZ and WAZ were not significantly different between the two groups (mental and non-mental disabilities). In fact, a study conducted in Nepal showed evidence of the increased risk of cognitive delay associated with severe stunting [21], which may negatively affect on the brain development in early life [22].

In any case, even though there have been efforts to cement a causal relationship between stunting and risk of disability, this has proven difficult due to our lack of understanding of the underlying biological mechanisms for childhood mental and physical disability, likely processes controlled by multiple factors [21]. However, it should be also noted that in developing countries, with various degrees and types of malnutrition being common among the entire population [23,24], prevalence of problems on the nutritional status in disabled children could be inevitably higher than the general population [18,25].

Consistent with this, our findings show that HAZ was significantly lower in the children with a physical disability (*t* = 3.94, *p* < 0.01). Further, the mean of WAZ (−2.90) classifies them as underweight, even when WAZ was not significantly different between the two groups (disabled and not disabled). At the same time, the mean of HAZ (−3.22) classifies these children as severely stunted. Similar evidence was found in a study in Iran where the anthropometric assessment showed higher prevalence of malnutrition in disabled children compared to non-disabled children, with the prevalence of stunting being remarkably higher in physically disabled children [25]. These authors ensure that a limited range of motion (a characteristic of physically disabled children) has a much lower lean body mass (LBM). Reduced LBM is also known to be one of the negative functional repercussions of stunting in general [26,27]. As well as the children with mental disabilities, physically disabled children have malnutrition due to feeding problems, but this could also be caused by several other reasons [28]. These unknown reasons would be difficult to determine in our sample given the miscellaneous nature of the physical disabilities of these children. In our study, Bal Griha includes children with disabilities on the sample that could also contribute to increase the prevalence of stunting, given the relationship between poor nutritional status and disability [17,18,21,25,29]. It should also be noted that, in general, children who are not adequately nourished are at risk of failing to reach their developmental potential in cognitive, motor, and socioemotional abilities; two of the key risk factors of this poor development are the severe underweight and stunting [22]. This suggests that underweight or/and stunting could play two different roles in disabilities, they could be a consequence of the disability, but they could also be one of the risk factors of developing a disability.

Regarding the sex, WAZ was significantly lower http://www.unicef.org/publications/files/Africas_Orphaned_and_Vulnerable_Generations_Children_Affected_by_AIDS.pdf among boys (*t* = 2.62, *p* < 0.05). The mean of WAZ (−2.37) classifies boys as underweight. It should also be taken into account that the evaluation of a disabled child’s weight may not be accurate seeing as there is no adjusted weight standards available; in fact, studies on disabled children normally use non-disabled healthy children’s charts or references [30]. However, a recent study that evaluates the effects of number and sex of siblings on malnutrition of children under 5 years old in Bangladesh, India, and Nepal, reports that a different feeding treatment for girls relative to boys (based on number and sex of siblings) may be occurring in South Asia, advantaging the food access for boys over girls [31]. The same study showed that girls with three or more sisters were at significantly greater risk for severe underweight, where no effect is seen for boys. The differences in the treatment as one of the causes of higher risk malnutrition could not be applicable in this study since differences between sex were not observed in the orphanages. Going back to the Nepal Demographic Health Survey from 2011, we observed that boys under 5 years old are slightly more likely to be underweight (30%) than girls under 5 years old (28%) [16]. Opposite to this, a different study conducted in Nepal with former Kamaya families, who are among the most socioeconomically disadvantaged groups in Nepal [32], showed that girls were more likely to be underweight [33]. This could be due to the sex differences on the treatment mentioned above. However, in a study where the range of age is more like this study (6–10 years old), the prevalence of underweight was slightly higher in boys (52.46%) than in girls (46.09%) [34].

A parallel problem to this study is knowing the prevalence data in Nepal because the current information is very limited. Recently, Bathia et al. [35] proposed a mixed methods study, including large-scale surveys, case data from the police, court system, newspapers, community consultations, and child participation, which can help to know the reality of a situation that affects the child population.

Limitations in our study included the small sample population and the limited reach of the sampling process to the frailest and most vulnerable of children in Nepali orphanages.

## 5. Conclusions

Evidence of an association between undernutrition and mental and physical disability (wasting and stunting, respectively) was found in this study. There is also weak but significant evidence of a relationship between underweight and being male. Different types and degrees of undernutrition were significantly related with the presence of the disability in institutionalized children. More research about nutritional status of institutionalized disabled children would be necessary to shed more light on their situation, and the fact of being a disabled child living in an institution increasing the vulnerability of poor health conditions. The design and implementation of specific interventions would also contribute to improve their quality of life.

## Figures and Tables

**Table 1:** Characteristics of the sample. Mean and standard deviation of age, height, weight, and growth-related characteristics.

	Mean	Standard Deviation (SD)
Age (years)	10.4	3.4
Height (cm)	128.7	18.7
Weight (kg)	26.0	10.1
WAZ	−1.9	0.9
HAZ	−1.9	1.2
BAZ	−1.4	1.4

WAZ = Weight-for-age Z-score, HAZ = Height-for-age Z-score, BAZ = Body mass index (BMI) for age.

**Table 2:** Mean difference (and standard deviation; SD) for WAZ, HAZ, and BAZ compared with the WHO’s standard for age and grouped by orphanage, physical disability, mental disability, and sex.

ORPHANAGE	BAL GRIHA 1 (N = 24)	BAL GRIHA 2 (N = 23)	T	SIG
mean difference (SD) of WAZ	−2.03 (0.84)	−1.87 (0.90)	−0.44	0.663
mean difference (SD) of HAZ	−2.43 (1.20)	−1.33 (0.93)	−3.47 **	0.001
mean difference (SD) of BAZ	−1.26 (1.34)	−1.53 (1.50)	0.65	0.516
**PHYSICAL DISABILITY**	**NO (*n* = 39)**	**YES (*n* = 8)**	**T**	**Sig**
mean difference (SD) of WAZ	−1.85 (0.72)	−2.90 (2.02)	1.77	0.090
mean difference (SD) of HAZ	−1.62 (1.07)	−3.22 (0.85)	3.94 **	0.000
mean difference (SD) of BAZ	−1.43 (1.44)	−1.18 (1.42)	−0.46	0.650
**MENTAL DISABILITY**	**NO (*n* = 41)**	**YES (*n* = 6)**	**T**	**Sig**
mean difference (SD) of WAZ	−1.85 (0.96)	−2.92 (1.11)	1.75	0.094
mean difference (SD) of HAZ	−1.78 (1.07)	−2.62 (1.05)	1.62	0.111
mean difference (SD) of BAZ	−1.20 (1.40)	−2.70 (0.91)	2.53 *	0.015
**SEX**	**GIRLS (*n* = 24)**	**BOYS (*n* = 23)**	**T**	**Sig**
mean difference (SD) of WAZ	−1.54 (0.96)	−2.37 (1.11)	2.62 *	0.020
mean difference (SD) of HAZ	−1.88 (1.07)	−1.90 (1.05)	0.04	0.965
mean difference (SD) of BAZ	−1.12 (0.90)	−1.66 (1.14)	1.30	0.200

* *p* < 0.05; ** *p* < 0.01.
